# Information Connections among Multiple Investors: Evolutionary Local Patterns Revealed by Motifs

**DOI:** 10.1038/s41598-017-14141-1

**Published:** 2017-10-25

**Authors:** Qing Guan, Haizhong An, Nairong Liu, Feng An, Meihui Jiang

**Affiliations:** 1 School of Humanities and Economic Management, China University of Geosciences, Beijing, 100083 China; 2 Energy Systems Division, Argonne National Laboratory, Argonne, IL 60439 USA; 3 Key Laboratory of Carrying Capacity Assessment for Resource and Environment, Ministry of Land and Resources, Beijing, 100083 China; 4 Key Laboratory of Strategic Studies, Ministry of Land and Resources, Beijing, 100812 China

## Abstract

The concept of motifs provides a fresh perspective for studying local patterns, which is useful for understanding the essence of a network structure. However, few previous studies have focused on the evolutionary characteristics of weighted motifs while further considering participants’ differences. We study how information connections differ among multiple investors. The evolutionary 10-year trend of weighted 3-motifs in China**’**s energy stock markets is explored for the networks of co-holding behaviors among shareholders, who are classified as companies, funds and individuals. Our works allow us to detect the preferential local patterns distributed among different agents as their fluctuate involvement in networks. We find that the diversity of shareholders contributes to the statistical significance of local patterns, while homophily always exist among individuals. Modules of information connections are stable among reserved investors, which is especially apparent among companies. Individuals prefer to keep their connections with companies and funds. Unsteady modules happen owing to strengthen links among funds during the time that they are main participants in stock markets. More details about multiple investors informationally connected in evolutionary local patterns can be detected by our work.

## Introduction

How does one complete a puzzle? Apart from knowing the whole picture at first glance of the general graphical structure, the task requires much more attention to the blocks. In fact, although the information from each block is different, the shape of them normally can be classified into a limited number of types, which promotes the reconstruction of the whole puzzle. Similarly, motifs, which are defined as ‘recurring and significant patterns of interconnections’^1^, are the simple structural blocks of complex networks. It has been proven that motifs play essential roles in network evolution^2^ and optimization^3^. Considering multi agents into one unit, motifs are suitable for understanding financial behaviors among diverse investors, which is a long-standing issue for explaining herding effects in stock markets^4–6^. Theoretical analysis has approved the importance of information connections in diffusing signals about stocks that traders receive for making financial behaviors^7,8^. As a result, how investors are informationally connected has drawn large attention from researchers. To identify information links from realized trades^8^, common shareholdings have been widely adopted as a structural perspective to describe information connections among investors^9,10^. Since the necessity of considering investor heterogeneity^11^ into the studies of trading activities has been recognized, the problem of characterizing the distribution of information connections among diverse investors remains open. In this paper, we study how information connections differ among multiple investors, which is revealed by evolutionary weighted motifs.

The concept of motifs provides a platform to study local network patterns^12^, especially the interactions among agents. Since it was first proposed by Milo *et al*.^1^, the concept of motifs has grown in its importance^13–15^, and has been widely applied to international trade^16,17^, the internet^3^, transportation^18^, social contagion^19^, and biological systems^20,21^. Latent variable modeling^22^, which includes Gaussian process latent variable model^23^, t-distributed stochastic neighbor embedding^24^, locally linear embedding and so on, are introduced for motif-finding with quick speed and high quality. Graph-theories are combined for financial analysis on time series to reveal underlying connectivity structure^25,26^. These studies approve motifs’ function in different application fields. Instead of requiring the statistical significance, recent studies extend the definition of motifs as a class of equivalent sub-graphs^27^. Based on this more general concept, both for communication networks, Li *et al*.^28^ explore motifs’ temporal characteristics, while Kovanen *et al*.^29^ consider attributes such as sex and age to classify agents in motifs. They approve that more structural features can be revealed by the motifs with the classification of components. However, not much is known about evolving weighted motifs among multiple investors. In our study, evolutionary characteristics would be explored for the motifs with the classification of both agents and links.

Components classified motifs provide a microscopic perspective for understanding local information connections among multiple investors. In one aspect, investors with different financial attributes always have specific investment preferences^11^. Previous works have manifested their differences in responses to the stock information^30^, and financial interactions among a certain type of investors are studied^31,32^. In another aspect, there are significant differences in closeness among investors based on their investment similarities^7^ and geological closeness^33^. The weak tie theory^34^ makes the interaction among investors much more complex because of the tendency of receiving information signals beyond neighbors. Apart from social connections^4,35,36^, co-holding behaviors in the same listed companies’ stock are widely adopted to describe information connections in recent works^7–9^. It reflects investor shareholding similarities^37^, which leads to the formation of paths for information diffusion in a local pattern^38^. Pareek^8^ examined its effect on investors’ trading behaviors, which approves the networks feasibility. Li *et al*.^9^ measured the evolutionary stability for China’s listed energy companies by associated maximal connected sub-graphs from the perspective of global pattern. Apart from these valuable works with a global perspective, more details need to be explored by exploring local patterns. In this paper, we introduce weighted motifs to model investors of different types into one unit with consideration of their co-holding strength.

In our study, evolutionary weighted motifs and agents’ financial attributes are both considered to explore local co-holding patterns among shareholders. Considering the importance to stable society and economic development, Chinese listed companies and shareholders whose main business is energy are selected as the case, and the evolution period is from 2006 to 2015. After knowing the components in co-holding networks, we first detect weighted motifs and their involved multiple investors in 10 years. Correlative characteristics of motifs’ proportional fluctuation with the changes of network structures are also measured in this part. Further, quantitative researches with more economic interpretations are provided. Statistical importance of local patterns are gauged with the financial classification for agents to discover typical groups of information connected traders. At last, the evolving motifs among multiple investors are detected in both short and long term. This work aims at indicating the dynamic connecting modules as the constitutional fluctuation of different investors.

The remainder of this paper is organized as follows. The next section include the data source and models. Section 3 shows the results classified into six parts: network components, evolutionary motifs, their reflection on network structure, preferential local pattern, motifs’ significance and the local structural evolution. Finally, we discuss interesting findings and conclude the paper in Section 4.

## Data and Modeling

### Investment data

The source of our data is the Wind financial database (http://www.wind.com.cn/), which provides a professional platform for researchers with various statistical data for the Chinese financial market. Data of stock market can be divided according to the major business run by the listed companies. As a result, we downloaded the data from the Chinese energy stock market on September 14, 2016, including the code and name of Chinese energy listed companies. At the same time, the name of their top 10 circulation shareholders are obtained, which are the only ones that can be disclosed. Circulation shareholders are more fluctuant than un-circulation ones are. We select the time period from 2006 to 2015 to see the evolutionary trend of the basic structural units constructing co-shareholding relations among investors. For convenience, we give each shareholder a unique number following the letter ‘H’.

Based on the information about investors in our data, we classify shareholders into three categories: companies, funds and individuals^39^. Companies are the institutions which include both energy-related companies, such as Yanzhou Coal Mining Company Limited, and financial investment-related companies, such as Central Huijin Investment Limited. Shareholders, which are classified as funds, are always published by banks. Individuals are the retails actively joining the investment in stock markets.

### Co-holding networks and motifs

The motifs are applied to explore the local pattern characteristics based on co-holding behaviors among shareholders. As a result, in this section, we show our methodology from four parts. First, the construction of the complex network is described, which provides a measurement for the basic relations among shareholders. Second, the motifs in our work are introduced, showing all types of local patterns that we can explore from the complex network. Third, to explore the correlations between motifs and the network structure, statistical indicators for the network structure and their corresponding meanings are provided. Further, to quantify these correlations, the Pearson correlation coefficient is adopted for the measurement.

#### Network modeling

**Figure 1:**
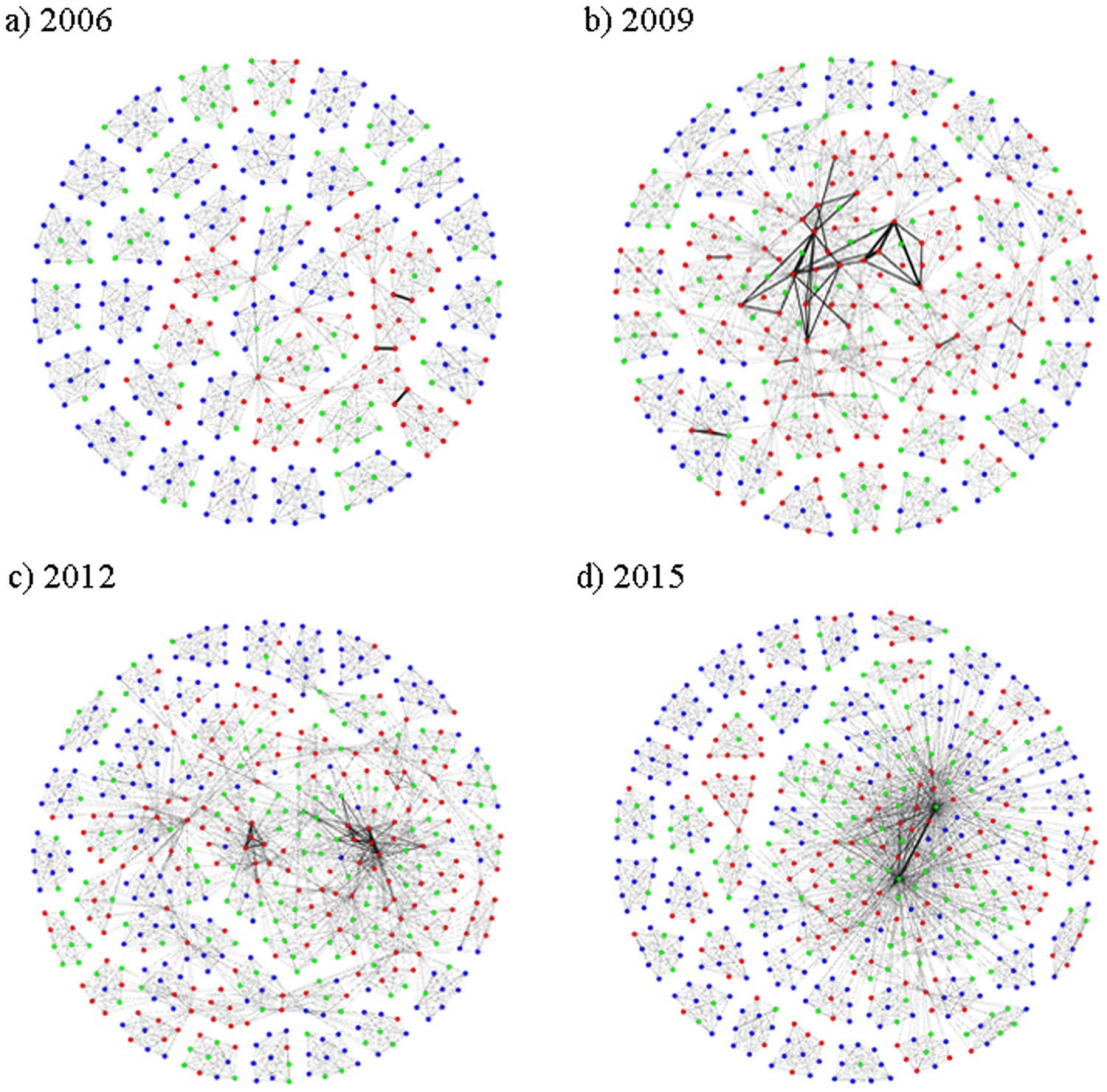
Networks for co-holding behaviors among shareholders in four years. Nodes are colored into three types: Greens are companies, reds are funds and blues are individuals.

A complex network is used to describe the relations among investors. The original data show the relations between Chinese energy listed companies and their corresponding top 10 circulation shareholders. In our studies, relations among investors are derived from the original data. As Li *et al*.^9^ did in their previous studies, we take shareholders as nodes, whether they take a stock share in the same listed company as the edge, and the number of listed companies they co-shareholding as the weight of the edge. That is, we construct a weighted but undirected network to describe the co-holding behaviors among investors. Using ${\rm{H}}=\{{H}_{1},{H}_{2},\cdots ,{H}_{m}\}$ to represent shareholders, the matrix A describes the co-holding relations as follows:

1 $${\rm{A}}=\{\begin{array}{c}{a}_{ij}={w}_{ij}\,{H}_{i}\,and\,{H}_{j}\,co-hold\,{w}_{ij}\,Chinese\,listed\,company\\ {a}_{ij}=0\,{H}_{i}\,and\,{H}_{j}\,co-hold\,no\,Chinese\,listed\,company\end{array}$$

where *w*_*ij*_ is the number of listed energy companies that shareholders *H*_*i*_ and *H*_*j*_ co-hold. Based on this definition, we construct 10 networks for each data unit from 2006 to 2015. Figure 1 shows the example networks for 2006, 2009, 2012 and 2015, where the nodes are shareholders and the links are the co-holding behaviors among them.

#### Motifs in co-holding networks

Motifs are the basic units of a network, so the recurrence of them contributes to the whole network^40^. Fluctuations of the number of motifs affect the network structure, whereas structural changes of the network pattern reflect changes in the motif constitutions. Studies on motifs in networks help us better understand the essence of the network structure^1^. According to the basic definition introduced by Milo *et al*.^1^, the recurring patterns of interconnections occurring in the real network at numbers that are significantly higher than in corresponding random network are called motifs^1^. That is, the subgraphes with low statistical significances always receive less attention from researchers. However, consideration of agents’ attribute into motifs need complete information extraction. As a result, in our study, the extended definition from Onnela *et al*.^41^ is adopted, and ‘motifs’ denote to equivalent subgraphes with independence of their statistical significance. 3-motifs are studied since they can model at most three different types of shareholders into one unit, which corresponds to our classification for investors. Each motif involves paths connecting three investors.

**Figure 2:**
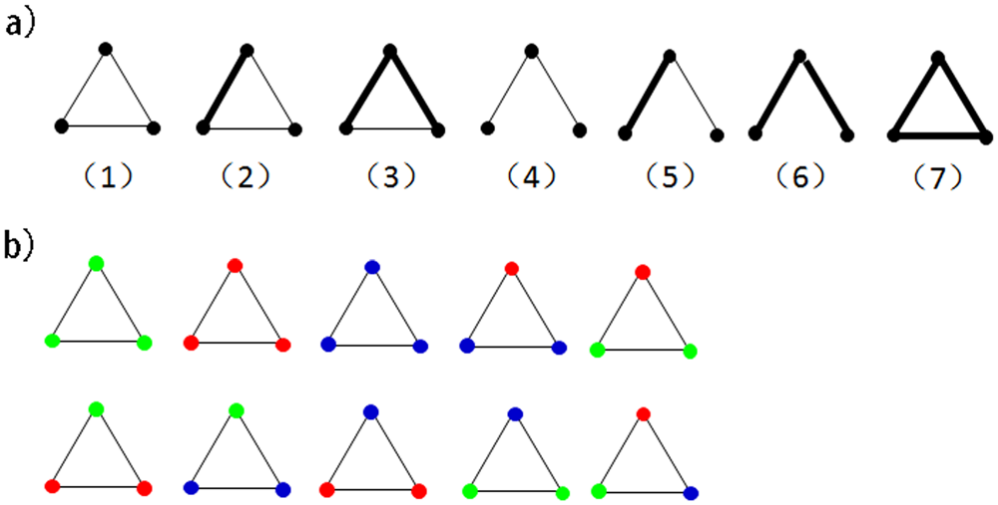
(**a**) Seven types of weighted triangle motifs. Thick lines as strong co-holding relations and thin lines as weak co-holding relations. (**b**) Motif 1 with colored investors: Greens are companies, reds are funds and blues are individuals.

Here, two levels of classification for links and nodes in 3-motifs are involved in this paper. First, the weight of connections are classified for 3-motifs, which are shown in Fig. 2(a). For the Chinese energy stock market, a very small number of shareholders co-hold shares in more than two of the same energy listed companies. Therefore, we classify links into two types: strong ones and weak ones^41^. Thick links in the figure indicate that there are many same listed companies held by two related shareholders, whereas light ones indicate that two shareholders co-hold only one common listed company. Among these bipartite weighted 3-motifs, motif 1, motif 2, motif 3 and motif 7 are the ones with triadic closure. Motif 4 are the open ones with only two connections among three agents. Further, the classification of investors are considered for each weighted 3-motif, and Fig. 2(b) shows an example based on the weighted motif 1 in Fig. 2(a).

**Figure 3:**
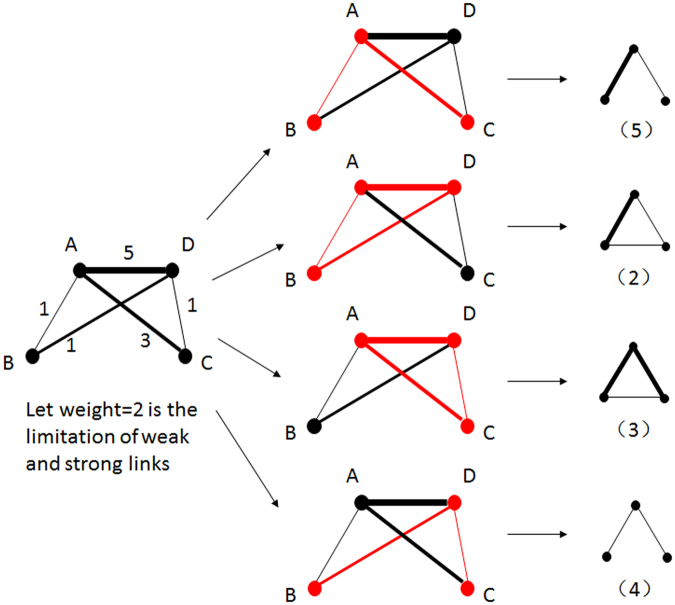
Schematic figure of the motif calculation.

These two levels of classification are used to explore local structural characteristics from different perspectives. Weighted 3-motifs provide general understanding for answering how investors are connected in co-holding networks. Further classification of nodes in 3-motifs provide specific exploration on information connections among multiple investors. It helps to answer how certain types of shareholders are connected. A schematic view of motif calculation is shown in Fig. 3.

#### Motifs with statistical significance

Detection of 3-motifs helps to explore information connections among multiple investors. In our study, we introduce Z-score^42,43^ into our study to measure the importance of 3-motifs in networks. It compares the statistical significance of certain motif in real networks than in the random networks. The basic definition of Z-score, which is widely used in undirected networks, is as follows,

2 $${Z}_{i}=\frac{{N}_{rea{l}_{i}}-{N}_{ran{d}_{i}}}{{\sigma }_{ran{d}_{i}}}$$

where ${N}_{{{real}}_{i}}$ is the number of motif i in real network. ${N}_{{{rand}}_{i}}$ is the number of motif i in random networks, $\langle {N}_{{{rand}}_{i}}\rangle$ is its average, and ${\sigma }_{{{rand}}_{i}}$ is its standard deviation. The motif with higher Z-score value is more important to the network.

For weighted networks, Z-score is promoted with the combination of motifs’ intensity^27,44^, coherence^27,44^, entropy^45^, within-module degree^46,47^ and so on. According to Onnela *et al*.^27^, the Z-score definitions for unweighted network and weighted network coincide for binary weights. Considering the fact that links’ weight in 3-motifs are classified into two types in this work, basic definition of Z-score is adopted to measure structural importance.

#### Indicators for measuring network structure

The constitution of triangle motifs is a significant reflection of the network structure. As a result, we calculate the indicator values for the global network pattern to see whether there are correlated characteristics between the evolution of network structure and motif composition. To measure the global network pattern, in addition to the number of nodes and edges, we select the following indicators: average weighted degree, network diameter, network density, modularity and average weighted clustering coefficient.

The average weighted degree (AWD) is used to describe each node’s connection with other nodes. A higher value of this indicator infers that most nodes in the network have a high correlation with other nodes with many strong links. The definition is as follows^48^:

3 $${S}^{AWD}=\frac{1}{|H|}$$

where H denotes the set of shareholders, t represents a shareholder belonging to H, and *S* ^*WD*^(*t*) is t’s weighted degree, which is further defined in this equation. *N*_*t*_ is the set of t’s neighbor nodes, k is t’s neighbor, and *w*_*tk*_ is the weight of link between t and k.

The network diameter (NDia) is the maximum of all of the nearest routes from one investor to another. Normally, if a network has a relatively low diameter, the relations among agents will be close, and information can be spread among investors over a small amount of time. The definition of network diameter is as follows^49^:

4 $${S}^{ND}=ma{x}_{t,v\in H}({L}_{min}(t,v))$$

where *t*, *v* are all shareholders belonging to H and *L*_*min*_ (*t*, *v*) is the smallest number of paths connecting t and v.

The network density (NDen) describes the scale of co-holding behaviors among shareholders. If the network density is large, the co-holding behavior will be a common phenomenon for investors. The measurement of these extensive relations is as follows^50^:

5 $${S}^{ND}=\frac{2n}{m(m-1)}$$

where m is is the number of shareholders, and n is the number of real edges.

The average weighted clustering coefficient (AWCC) is used to measure the local connection density. It describes whether each node’s neighbor nodes are closely connected with each other. Its definition is as follows^27^:

6 $${S}^{AWCC}=\frac{1}{|H|}{\sum }_{t=1}^{|H|}\frac{2{n}_{t}}{{N}_{t}({N}_{t}-1)}$$

where *n*_*t*_ is the number of real links among shareholder t’s neighbor investors.

Modularity is an indicator that classifies nodes into different communities according to their closeness. Normally, it is used to measure the linking density inside communities compared with the links between communities. The partition results are better when the modularity value is higher. As a result, this indicator helps determine whether agents have apparent differences in closeness with each other. The definition of modularity is as follows^51^:

7 $${\rm{Q}}=\frac{1}{2m}{\sum }_{i,j}[{w}_{ij}-\frac{{A}_{i}{A}_{j}}{2m}]\delta ({c}_{i},{c}_{j})$$

where w_ij_ is the weight of the link between i and j, A_i_ is the weighted degree of i, c_i_ is the community to which i is assigned, δ(c_i_, c_j_) is 1 if c_i_=c_j_ and 0 otherwise, and ${\rm{m}}=\frac{1}{2}{\sum }_{{\rm{i}},{\rm{j}}}{{\rm{w}}}_{\mathrm{ij}}$.

#### Correlation measurement

To answer whether there is correlative characteristics between the network structure and local patterns, we measure correlations between two fluctuation trends, which is motifs’ percentage and network indicators’ value over ten years. Here, we do not concern how motifs’ constitution contribute to the network structure. As a result, we try to grasp the significant characteristics by correlation coefficient. Previous studies have applied Pearson correlation, Kendall correlation^52^ and Spearman rank correlation^53^ for various research fields. These indexes have different consideration for data. Considering our low data size and the focus on correlative features, we adopt Pearson correlation in this work. As one of the most commonly used indicator, we select the Pearson correlation coefficient^54^ to study the evolution similarities between the network structures and the constitution of motifs from 2006 to 2015. That is, this indicator provides a general perspective to measure how two fluctuation trends are correlated. The coefficient is between −1 and 1, where 1 represents total positive correlation, 0 means no correlation, and −1 indicates total negative correlation.

In our work, the percentages of the seven weighted triangle motifs and seven global network indicators (the number of nodes, the number of edges, average weighted degree, network diameter, network density, modularity and average weighted clustering coefficient) are calculated for each data unit from 2006 to 2015. As a result, the evolutionary trend over 10 years for each motif and indicator can be obtained. The matrix of the Pearson correlation coefficient, R, is defined as follows:

8 $${\rm{R}}=[\begin{array}{ccc}{r}_{11} & \cdots  & {r}_{1N}\\ \vdots  & \ddots  & \vdots \\ {r}_{M1} & \cdots  & {r}_{MN}\end{array}]$$

where M is the number of motifs and N is the number of global structural indicators. Thus, M=N=7.

The equation for the Pearson correlation coefficient is as follows:

9 $${r}_{m,n}=\frac{{\sum }_{i=1}^{t}({x}_{i}^{m}-\overline{{x}^{m}})({y}_{i}^{n}-\overline{{y}^{n}})}{\sqrt{{\sum }_{i=1}^{t}{({x}_{i}^{m}-\overline{{x}^{m}})}^{2}}\sqrt{{\sum }_{i=1}^{t}{({y}_{i}^{n}-\overline{{y}^{n}})}^{2}}}$$

where *r*_*m, n*_ is the Pearson correlation coefficient between motif m and indicator n and t is the number of spot data for each variable. In our study, t=10 is the number of years. $\mathop{{x}^{m}}\limits^{\bar{} }$ is the average percentage value of motif m, which is the same as $\mathop{{y}^{n}}\limits^{\bar{} }$.

## Results and Analysis

### Components in co-holding networks

In this part, we explore basic components for co-holding networks to understand who are participating in the China energy stock market, and how they are connected. This work helps to interpret our results economically, and have basic knowledge of the market with consideration of investors’ financial attributes.

We track the scale of different investors for 10 networks respectively, and their percentages are shown in Table 1. Generally, in most years, the percentage of companies is around 30 with a relative steady trend. However, constitutions for funds and individuals are always opposite during these 10 years. From 2006 to 2009, although there is fluctuation, the percentage of funds is increasing while that for individuals is decreasing. Afterwards, both trends reversed, and changed their prominence in 2012. As a result, the scale fluctuation of three types of investors can be typically classified into three phases by the year 2009 and 2012.

**Table 1:** Percentage of each type of investors in each year (Unit: %).

	2006	2007	2008	2009	2010	2011	2012	2013	2014	2015
Companies	22.28	28.65	27.36	27.66	27.80	26.49	29.90	28.60	28.47	26.19
Funds	22.55	36.34	34.43	44.21	39.69	40.04	34.46	28.79	27.43	26.19
Individuals	55.17	35.01	38.21	28.13	32.51	33.47	35.64	42.62	44.10	47.62

**Figure 4:**
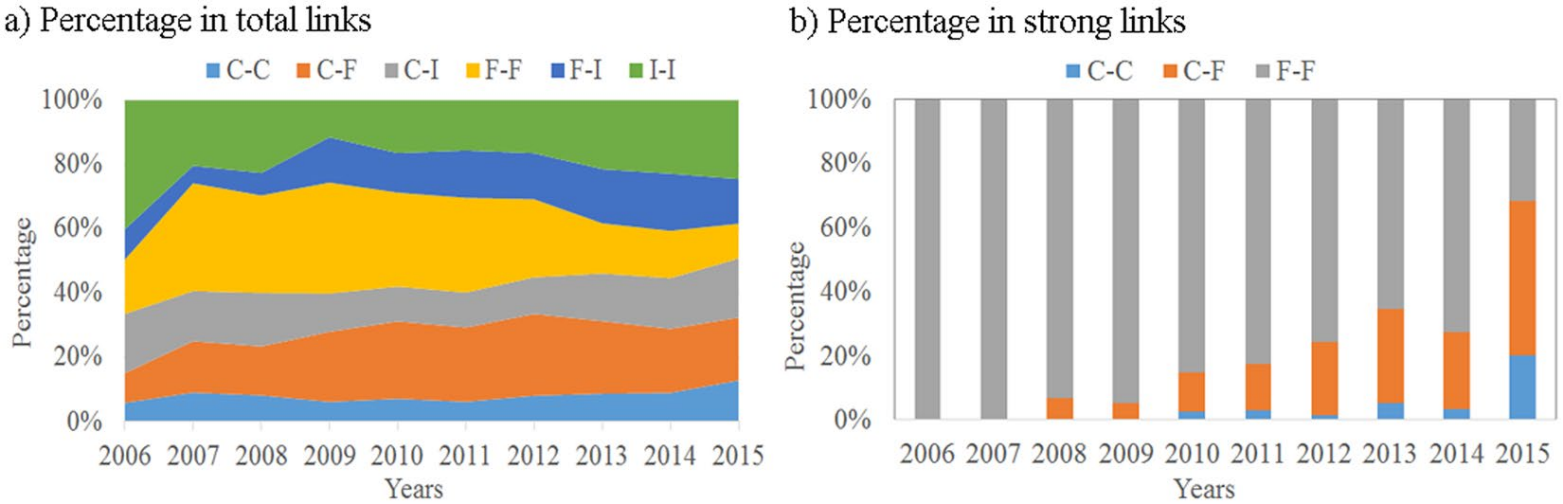
Percentage of links between pairs of shareholders with certain types. The percentage of shareholder pairs in certain financial types with (**a**) co-holding links or (**b**) thick links.

Further, we detect the distribution of co-holding relations among shareholders with different classified financial types. It helps to understand how shareholders are linked with each other based on their investment similarities. Figure 4(a) shows the percentage of co-holding links between shareholders with certain financial’ types in each year. With the consideration of linking strength, for each year, we further count the percentage of these shareholder pairs in the strong links, which is shown in Fig. 4(b).

From Fig. 4(a), we find that the distribution of co-holding relations has been equalized in recent two years. During the early times, there are high percentage of funds co-holding stocks with themselves. This trend has been decreased as the increase of co-holding relations between companies and funds. However, although strong links account a very little proportion in total co-holding links (the highest proportion is 2.78% in 2015), the distribution of them has apparent preference. Figure 4(b) approves that most strong links happen among funds, and it is relieved only in recent years as the rising participation of companies. For companies, our results show that they have expansion investment on energy listed companies. These results also reveal the investment limitation of individuals. Although they are always the main participants, their stocks always in a low number of limited companies. On the contrary, funds in the Chinese stock market come from certain number of banks. As a result, the funds from same banks would perform the overlap of their investment.

The constitution of components shows a fluctuant stock market. The involvement of multiple investors differs in each year, and their co-holding behaviors are unevenly distributed. This situation asks for an evolutionary perspective to study the local information connections among multiple investors. At the same time, considering the close relations between motifs and network structure, the works in this part provide references for explaining motif-related results in latter sections.

### Constitution of 3-motifs

**Figure 5:**
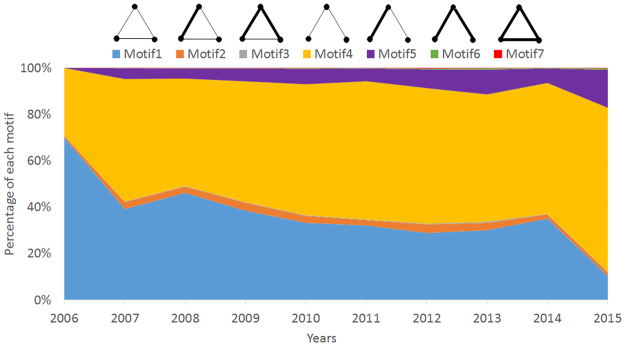
Evolution of each type of weighted triangle motif measured by their percentage in co-holding networks, from 2006 to 2015.

Constitution of motifs help to reveal the basic local co-holding patterns among shareholders. In this part, without the consideration of statistical significance, we explore all seven weighted triangle motifs for the Chinese energy stock market from 2006 to 2015. Here, investors are not classified in order to know general existence of motifs in co-holding networks. To compare the evolution of each type of motifs, we calculate their percentages in a total of seven types of motifs but not the numbers. Figure 5 provides the results.

The results show an apparent trend of the motif constitution over these 10 years in Chinese energy stock market. Networks are mainly composed by motif 1 and motif 4. As the steady increasing proportion of motif 4, motif 1 counts a decreasing percentage. The constitution reveals that the relations based on co-holding behaviors widely exist, but the connections are weak. However, in recent years, more couples of investors appear stronger shareholding overlaps. The more distinct rising trend of motif 5 than motif 2 approves that limited strong links always happen in the local pattern with less closely connections. In general, nodes in motif 1 can reach each other within one path. However, in motif 4, its structure looks like a tree being rooted with an intermediate agent, and two leaf-like nodes have to reach each other through 2 paths. Since the average linking cost for open motifs is higher than closed motifs, the proportional growth of motif 4 means a more scattered network structure over years, and the interaction of investment behavior would be slower.

Based on the above results, the local patterns for Chinese energy stock market exhibit apparent changes and fluctuation. In recent years, the co-holding behavior has gotten stronger, but stable relations have decreased, which has led to much higher indirect and long spreading paths.

### Associative characteristics with network patterns

**Figure 6:**
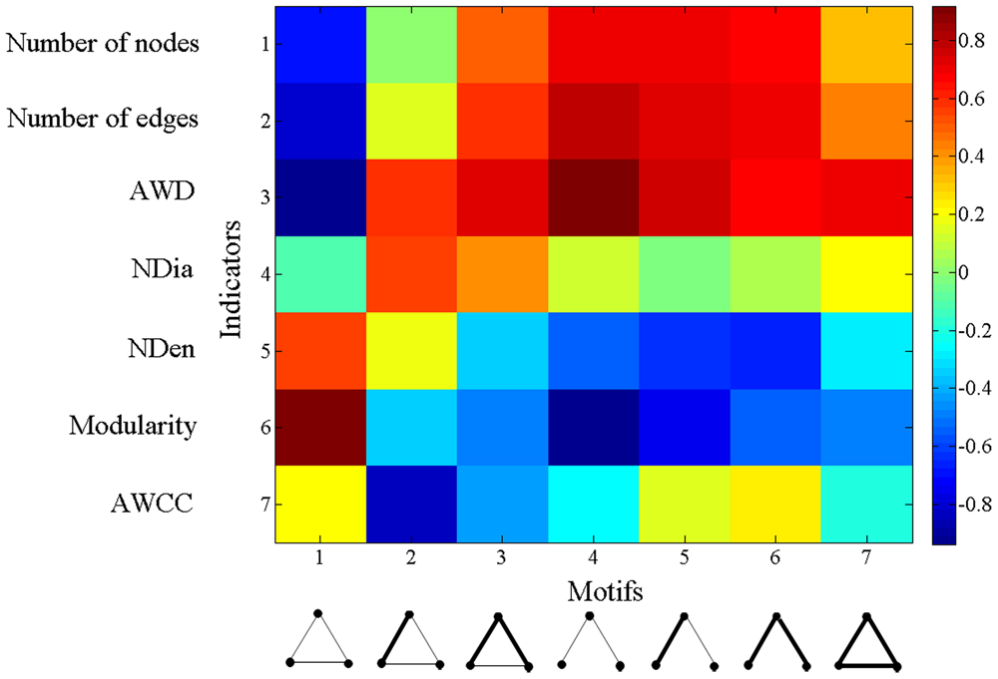
Pearson correlation coefficient (in each block) between the 10-years percentage fluctuation of each motif and the value fluctuation of each structural indicator.

The changes of network structure is the reflection of the fluctuation of local co-holding patterns. It is hard to quantitatively describe their bilateral correlations, but the correlation of their fluctuation trend can reveal their associated characteristics. Patterns of co-holding networks are described by statistical indicators from different perspectives. The results have been shown in Fig. 6. Each block represents the Pearson correlation coefficient between the motif proportion and the indicator value during 10 years. The colors provide intuition for our results, and their meanings are shown in the legend. Dark red is a fully positive correlation, and dark blue represents a fully negative correlation.

Corresponding to the motif constitution, network scales show a negative correlation with motif 1, but a positive relation with all four motifs from 3 to 6. Conversely, the modularity of networks appear to show opposite reactions. Although motif 3 is also a closed one with two stronger links than motif 1, its reflection on networks are closer with open motifs but not motif 1.

Results are correlated with the characteristics of stock market, depending on where these local patterns occur in networks. We find that motif 1 exists in every cluster, while motif 4 tends to occur in ‘core’ and across clusters. Taken the network structures shown in Fig. 1 as references, networks are composed by separate clusters and the ‘core’. Separate clusters show co-holding behaviors happen among the ten disclosed shareholders who hold stocks in the same listed company. ‘Core’ is the subgraph involving connected clusters, which means at least one associate shareholder holds stocks in more than two listed companies. However, there are always limited shareholders bridging the cross-cluster correlations. As a result, other agents in two clusters, together with the bridge-like nodes as the intermediate, form large numbers of open local patterns.

Networks in recent years show much denser cores than in 2006^9^. It means that shareholders tend to expand their investment to more selections with rising investment similarities, which is reflected by the positive correlation with network scales. In addition, the integration with more clusters means weakening boundaries among them, which leads to the negative correlation with network modularity.

As a result, increasing shareholders in Chinese energy stock market have investment expansion. There are higher investment similarities in local patterns, promoting higher global network scale and lower classification among them.

### Multiple investors in 3-motifs

**Figure 7:**
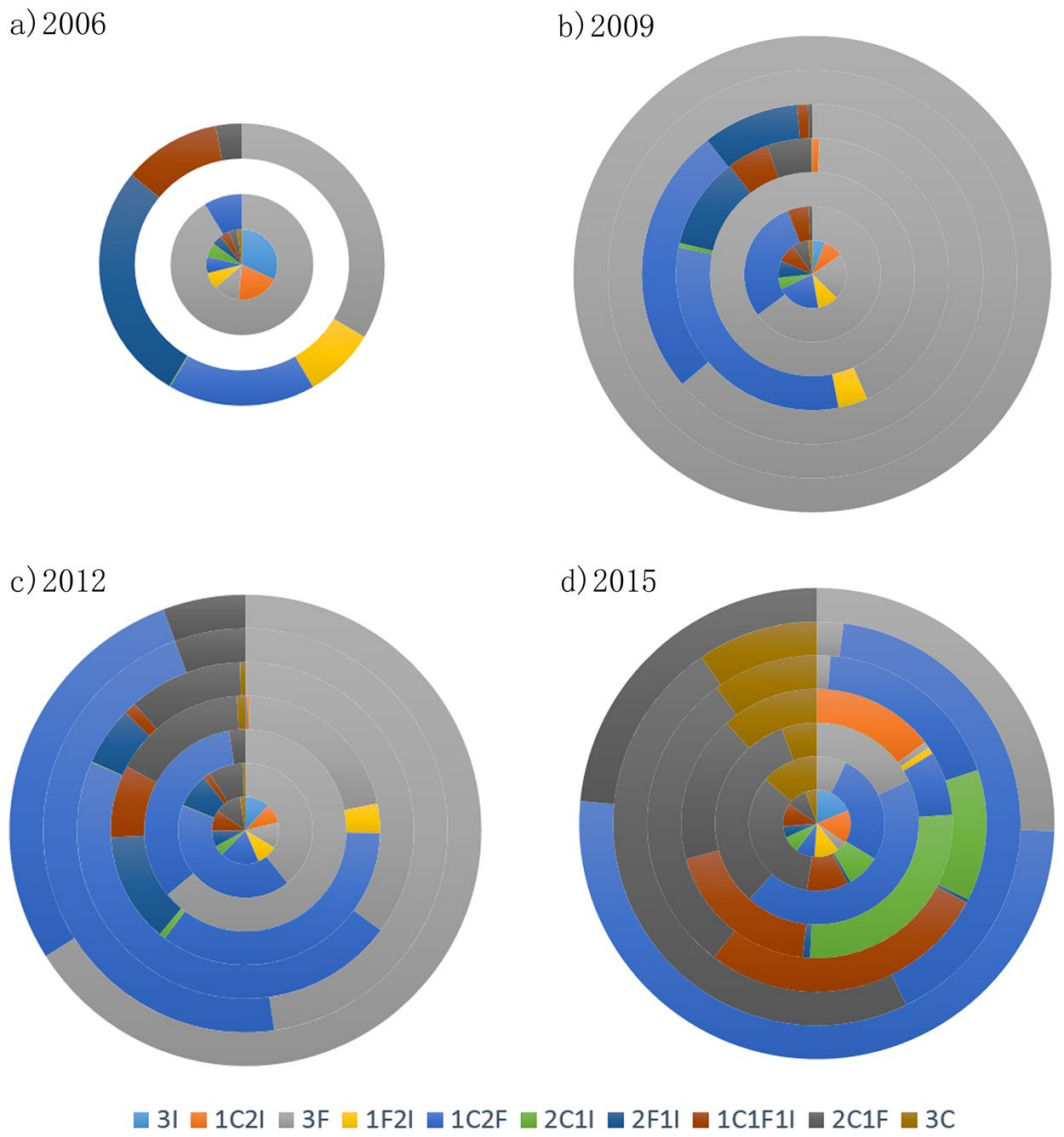
Combinations of three shareholders’ categories in each motifs (C: Companies; F: Funds; I: Individuals).

To understand how groups of different shareholders join in 3-motifs, we track the percentage of motifs with different combination of three shareholders’ financial types. Based on the three categories that we have classified for investors, there are normally 10 combinations for each motif, as shown in the legend of Fig. 7, where C represents companies, F is funds and I is individuals. The results in 2006, 2009 2012 and 2015 are selected as examples to show the evolution characteristics, which are shown as doughnuts in Fig. 7. The innermost circle is motif 1, whereas the outer donuts are sequentially from motif 2 to motif 7.

In 2006, only motifs 1, 2 and 4 are found in the network, and all seven motifs can be found in three other years.

For motif 1 and motif 4, the combination of multiple investors are much more abundant than other motifs. Especially, investors involved in motif 3, motif 6 and motif 7 are mainly from certain groups of investors with certain financial types. For example, in 2009 and 2012, funds are the main participants in these three motifs. However, in 2015, companies involved in the local connections with at least two strong co-holding relations. Except motif 1, the changing of main participants around 2015 also happen to three other motifs. In particular, individual join in motif 4 and motif 5 with companies or funds together. In comparison, combinations of multiple investors contribute more evenly in recent years.

These results help to explain how investors in three different types informationally connected with local structural characteristics. Moreover, how investors involve in different types of motifs are also answered.

### Statistical significance of 3-motifs

Although some motifs are high detected in networks, whether they are specific local patterns in the empirical stock market still need measurement. As a result, in this part, statistical significance are measured for each 3-motifs with multiple investors. Here, investors’ location differences in each weighted 3-motif are considered to classify their roles in connections.

**Figure 8:**
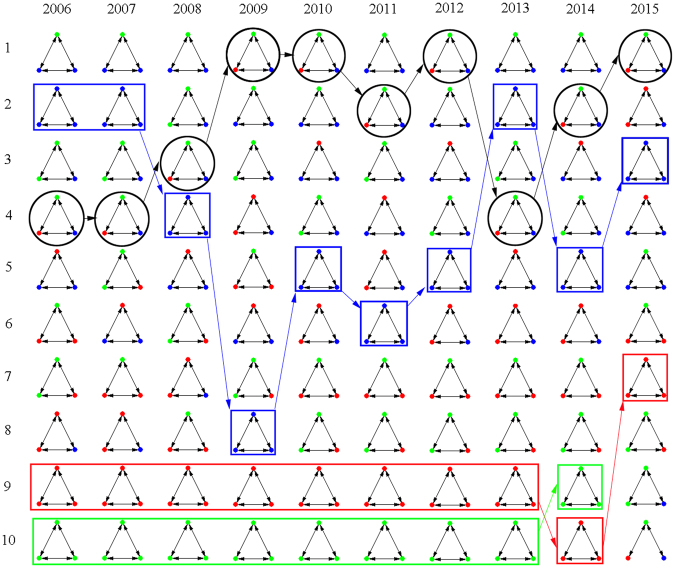
Top 10 motifs with highest Z-scores in each year, from 2006 to 2015. Green nodes are companies, red ones are funds, and blue ones are individuals. Black circles specify the motifs among agents with total different types. Squares specifies the motifs with three same type shareholders.

We calculate the Z-score for each classified motif, rank them from the maximum to the minimum, and select the top 10 motifs in each year. Figure 8 shows the results. Black circles specify the motifs among agents with total different types, while colored squares are used to highlight the ones among same type shareholders. The color of the square represent a certain type of shareholder: Greens are companies, reds are funds, and blues are individuals.

Whether shareholders tend to appear investment similarities with those who have similar financial attributes is answered by results with homophily and heterology characteristics. Results show that there are both homophily and heterology in motifs. In most years, heterology plays more significant roles than homophily. For the heterology, shareholders in motifs always appear with relative heterology (two agents with same type and the third one with different type) or with total heterology (agents with three different types). Especially, totally heterogeneous motifs experience fluctuations, but increasing importance in networks. In comparison, homophily exists in networks, but perform high uncertainty. What is more, the importance of homophily always appear among individuals. Although homogenous motifs exist among companies and funds, their importance in networks are much lower than the ones among individuals.

In conclusion, motifs show both homophily and heterology among shareholders in the Chinese energy stock market. Heterology contributes more to the networks than homophily. In addition, homophily always appear among individuals with higher importance.

### Evolution of local co-holding behaviors

**Figure 9:**
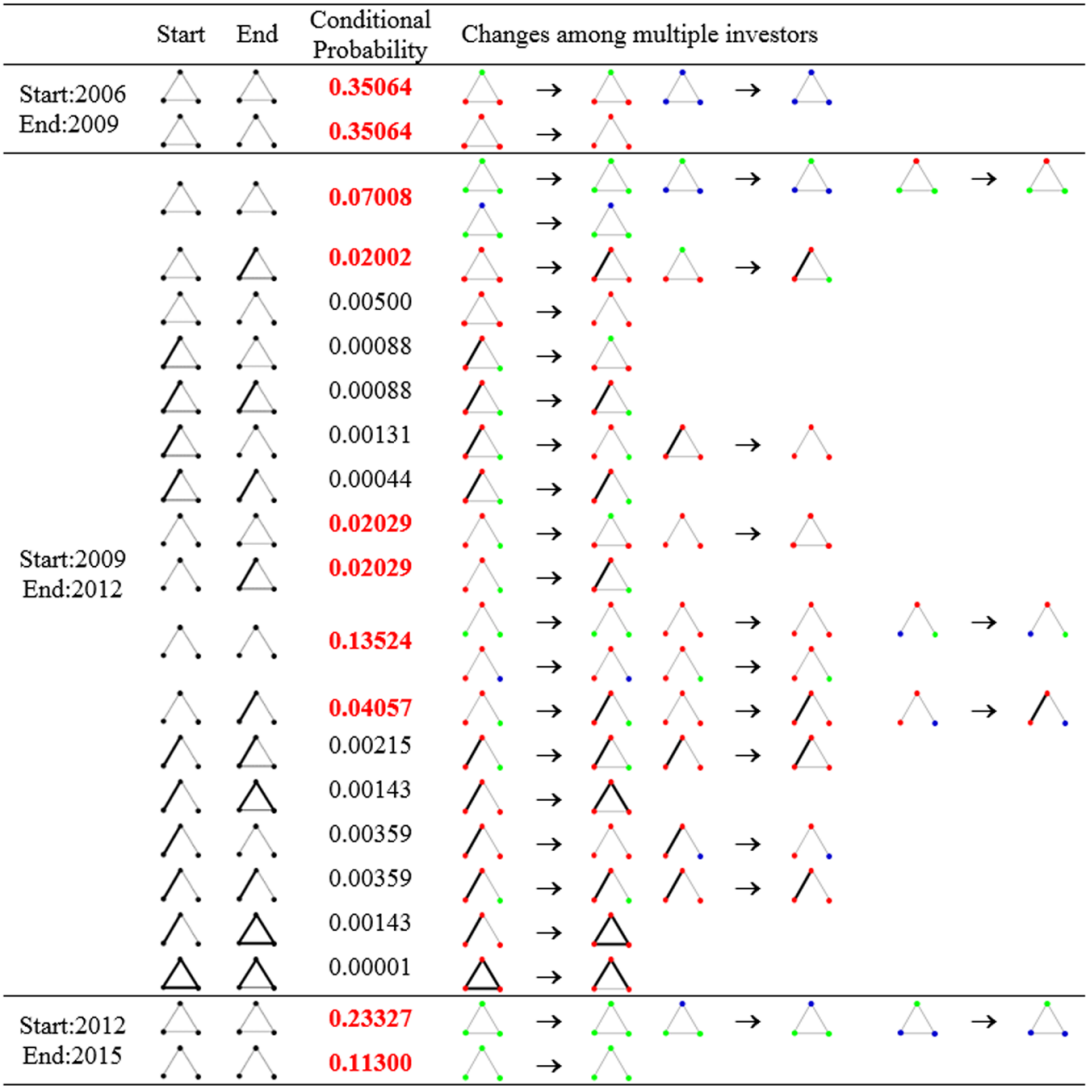
Changes of 3-motifs among overlap shareholders, 3-years term. Green nodes are companies, reds are funds, and blues are individuals. Red boldface are the conditional probabilities over 0.01.

In this part, we explore the dynamic changes of motif structure for each groups of agents. For Chinese energy stock market, the set of shareholders has high uncertainty, so there are limited investors reserved from each previous year. As a result, we find the overlap shareholders in the start year and the end year firstly, and then follow the motif structure changes among each group of three overlapped shareholders. Short term (1-year) and long term (3-years) are taken as the time expansion, and results are shown in Table 2 and Fig. 9 respectively.

**Table 2:** Changes of 3-motifs from the start module to the end module among overlap shareholders, 1-year term from the start year to the end year. Conditional probabilities over 0.05 is highlighted with boldface. Motif 1 is ; motif 2 is ; motif 4 is ; motif 5 is .

Start		2006	2007	2008	2009	2010	2011	2012	2013	2014
End		2007	2008	2009	2010	2011	2012	2013	2014	2015
Start	End									
1	1	**0.52596**	**0.33265**	**0.33400**	**0.35131**	**0.29899**	**0.29218**	**0.26711**	**0.28796**	**0.34987**
1	2	0.00000	0.02132	0.01066	0.00803	0.01260	0.01133	0.00959	0.00532	0.00122
1	4	**0.17532**	0.02843	**0.08883**	0.02008	0.01856	0.01670	0.00664	0.00760	0.00000
1	5	0.00000	0.00569	0.02843	0.00602	0.00265	0.00060	0.00443	0.00000	0.00000
2	1	0.00000	0.01882	0.00175	0.00644	0.00312	0.00176	0.00532	0.00413	0.00597
2	2	0.00360	0.00099	0.00962	0.01041	0.01585	0.01055	0.01452	0.01593	0.00597
2	4	0.00000	0.00793	0.01049	0.00793	0.00338	0.00477	0.00871	0.00767	0.00299
2	5	0.00360	0.00000	0.00262	0.00595	0.00572	0.00427	0.00750	0.00383	0.00000
4	1	0.00000	0.04224	0.04804	0.03893	0.01502	0.01706	0.00612	0.00707	0.02880
4	2	0.00000	0.03989	0.01747	0.01946	0.01567	0.03344	0.01311	0.00809	0.00000
4	4	**0.29152**	**0.36137**	**0.33189**	**0.38686**	**0.49174**	**0.47640**	**0.50516**	**0.50633**	**0.52984**
4	5	0.00000	**0.07978**	**0.06114**	**0.06813**	0.03918	**0.06143**	**0.05681**	0.02729	0.00576
5	1	0.00000	0.00000	0.00406	0.00316	0.00155	0.00229	0.00149	0.00303	0.00000
5	2	0.00000	0.00000	0.00947	0.00394	0.00802	0.00719	0.00298	0.00692	0.00522
5	4	0.00000	0.03048	0.01623	0.02603	0.01603	0.01602	0.02892	0.04626	0.04174
5	5	0.00000	0.01255	0.01217	0.01578	0.03776	0.02550	0.04084	0.04798	0.01565

Reserved local pattern refers to the condition that the motif established among certain three shareholders in the start year still exist in the end year without the consideration of their structural changes. Over these 10 years, we find that increasing connections among certain three shareholders can still be found in the next years. For example, almost 50% of the groups of shareholders from 2014 resolved their local pattern in 2015. Among these reserved local patterns, we calculate the conditional probabilities for each structural change, which is also shown in Table 2. Because the percentage of motif 3, 6 and 7 are too low to be counted, we only provide the changes among motif 1, 2, 4 and 5 here, which are also main motifs in 10 co-holding networks. The highlighted numbers with boldface are the relative high possibilities with the threshold of 0.05.

Results reveal that most motifs keep their style in a new year. However, we can find that, in early years, closed motifs with fully connections decomposed to open ones. In recent years, weak open motifs has involved more strong links. As a result, the decomposition of motifs do not mainly come from the closed ones which existed in networks. The increasing of co-holding strength contributes to the network fluctuation.

We further prolong the term for detecting motif changes. 3-years expansion is considered for the reason that companies, funds and individuals show three phases of proportional evolution in a longer term (as shown in Table 1). As the increasing of the time period, there are few triadic connections existent among limited overlap investors. Figure 9 shows all detected changes from the former motif at the start year to the latter motif at the end year. Involved investors are colored to show their position in each motif.

In a longer term, local patterns among companies are always steady, keeping their original connections. Stable structures are also reserved if a fund or an individual connects with two companies. At the same time, from 2009 to 2012, many motifs tend to get strengthened, which is mainly from the strong links established among funds. In comparison, as the decreasing proportion of individuals in the stock market, connections among themselves have turned to with companies. Increasing percentage of funds doesn’t contributes to steady structural evolution, but promote motifs being strengthened ones.

In this part, we demonstrate how local patterns are evolving among multiple shareholders. Multiple investors participate in the motif changes with their own roles with dynamic stock market in different time periods.

## Discussion and Conclusion

The evolutionary 3-motifs with classified agents reveal that information connections differ among multiple investors in the stock markets. In this paper, networks are modeled based on common holding behaviors among shareholders, who are classified into three categories: companies, funds and individuals. Weighted 3-motifs are detected from networks. Their statistical significance and dynamics are further measured. Main conclusions and discussions are as follows:

First, the detection on motifs is able to reveal preferential local patterns among diverse agents. In China energy stock market, individuals and funds appear opposite involvements, and high shareholding similarities mainly distribute among companies and funds. During 10 years, increasing proportions of shareholders are linked indirectly in local patterns without fully connections. At the same time, network structure shows a growth of network scale but lower modularity. Although motifs distribute more evenly among different groups of shareholders, companies and funds are main contributors for the motifs involving strengthen links.

Second, our study approves that heterology exists among traders in stock markets. Homophily shows its limit existence among individuals. It provides the research support for previous studies that focus on the social, geological and professional similarities among individuals. This paper shows the heterology with higher statistical significance than homophily in stock markets. Information would be more diverse from traders with different types. As a result, companies, funds and individuals tend to be informationally connected with each other as small groups.

Third, motif evolution reflect agents’ roles in dynamic local connections. Among the investors who have relative steady shareholdings, they tend to keep local patterns in both short and long terms. It is more common among companies, and individuals also tend to be steadily connected with companies. Unsteady local structure mainly come from funds because of strengthen connections established among themselves.

Our work is essential for understanding the relations between investors’ behaviors and the co-holding network structure. In particular, the results help to make the network reconstruction possible. As a result, our study will be of interest to researchers or stock managers who are interested in knowing more about investors’ behaviors. In the present work, we classify the weak and strong links based on a simple baseline, which will become a more precise classification in our future work.
